# Oxygen Equilibration Dynamics in Assisted Reproductive Technology Embryo Culture Media

**DOI:** 10.21203/rs.3.rs-8143184/v1

**Published:** 2025-12-16

**Authors:** Sanjana Kulkarni, Bailey K Morris, Sacha A Krieg, Thomas O’Leary, Adam Krieg

**Affiliations:** Oregon Health and Science University; Oregon Health and Science University; Oregon Health and Science University; Oregon Health and Science University

**Keywords:** ART Media Preparation, Reactive Oxygen Species, Oxygen Saturation Equilibration, Media Equilibration

## Abstract

**Purpose:**

Optimal oxygen tension is essential for successful *in vitro* embryo culture in assisted reproductive technology (ART). Reduced oxygen levels (3–8%) improve embryo development by minimizing oxidative stress, however, limited knowledge exists about transient oxygen fluctuations during handling outside hypoxic incubators. This study aimed to quantify the kinetics of oxygen equilibration in embryo culture media under conditions designed to mimic common ART laboratory practices.

**Methods:**

Embryo culture media droplets were prepared in room air and overlayed with light or heavy mineral oil. Dishes were equilibrated in a hypoxia chamber (5% O_2_, 5% CO_2_, 37°C), then transferred to an atmospheric incubator (18–19% O_2_, 5% CO_2_) for equilibration, and then this was repeated once more. Oxygen saturation was measured every 30 seconds using a fiber optic microprobe (PreSens, GmbH). Each experiment was replicated three times, yielding six equilibration and six reoxygenation curves. Data were fit to single-phase exponential models to calculate half-lives and rate constants.

**Results:**

Media equilibrated from atmospheric to hypoxic conditions within 12 hours. Light oil overlays demonstrated faster equilibration (half-life 71 min) compared to heavy oil (half-life 116 min, *p* < 0.0001). Upon reoxygenation, oxygen saturation rose rapidly, with light oil droplets reoxygenating with a doubling time of 50 min and heavy oil in 78 min (p < 0.0001).

**Conclusion:**

In ART media, hypoxic oxygen equilibration is a gradual process while reoxygenation is rapid. Oil viscosity significantly influences oxygen equilibration dynamics, with light oil permitting faster equilibration and reoxygenation. These findings underscore the importance of minimizing atmospheric exposure during routine handling and highlight the role of overlay oil in reducing transient oxygen fluctuations.

## Introduction

The success of *in vitro* embryo culture in assisted reproductive technology (ART) is critically dependent on the microenvironmental conditions to which embryos are exposed. A key component of this environment is oxygen, which plays a pivotal role in supporting embryonic development. *In vivo*, prior to implantation, embryos develop within the oviduct and uterus where the oxygen levels are considerably lower than atmospheric levels of 20–21% O_2_ [1, 2]. The physiological oxygen concentration in the female reproductive tract between ranges from 2% to 8% O_2_ [3, 4]. ART laboratories have adopted reduced oxygen culture systems, most commonly around 5% O_2,_ to more closely mimic the environment of the female reproductive tract [5, 6].

Excessive oxygen exposure during cell culture has been implicated in impaired embryonic development through oxidative mechanisms [7]. At high oxygen tension (approximately 21% O_2_), reactive oxygen species (ROS) are generated, leading to lipid peroxidation, protein modification, and DNA damage that ultimately compromise embryo viability [8, 9]. Early studies published between 1969 and 1971 demonstrated that culturing mouse embryos in low oxygen improved viability [10, 11]. Studies evaluating other species such as human, bovine, mouse, goat and pig have consistently shown that culture under hypoxic conditions reduces ROS generation, preserves mitochondrial integrity, and improves blastocyst formation and embryo morphology [12–16]. More recent studies in rhesus macaques have similarly reported that low-oxygen conditions (3–5% O_2_) support follicle growth, survival, and sustained steroidogenic function *in vitro* compared with atmospheric oxygen [17].

In human embryo culture, the use of low oxygen tension to improve ART outcomes is generally well accepted. Several randomized controlled trials and meta-analyses have demonstrated improved blastocyst development, implantation, and cumulative live birth rates in human embryos cultured under 5% oxygen compared with atmospheric oxygen [18, 19]. As a result, hypoxic incubation has become more common in many ART laboratories [20]. Despite understanding the importance of hypoxic environments in embryo culture, embryo manipulation often requires exposure to atmospheric conditions. The dynamic nature of oxygen exposure during routine laboratory handling remains poorly understood. While incubators maintain stable hypoxic conditions, embryos are transiently exposed to atmospheric oxygen during key manipulations such as gamete handling, fertilization checks, embryo assessments, and media preparation. These brief exposures may introduce ROS and undermine the protective effects of a low-oxygen environment. A fundamental gap exists in understanding how rapidly oxygen levels change when culture media transitions between atmospheric and hypoxic environments. The rate of oxygen equilibrium could impact embryos or oocytes as rapid reoxygenation may lead to prolonged exposure to nonphysiological oxygen concentrations, potentially affecting development and implantation.

The objective of this study is to quantify the time required for ART culture media to equilibrate from atmospheric (21% O_2_) to hypoxic (5% O_2_) conditions and vice versa. To our knowledge, this is the first study to systematically quantify the oxygen equilibration dynamics in ART culture media. By characterizing these equilibration times, we aim to provide practical data that may inform laboratory workflows and help minimize potentially harmful oxygen fluctuations during embryo culture.

## Methods/Material

### Preparation of culture droplets

Dishes were prepared at atmospheric oxygen levels at approximately 60 meters above sea level and at room temperature in a Baker Sterigard class II A/B3 biological safety cabinet under aseptic conditions. Embryo culture medium (GlobalTotal) was dispensed at room temperature into 60 mm polystyrene dish (Falcon 351007) in 30 μl sized droplets, 4 microdrops in each dish to mimic standard clinical embryology conditions. After the microdrops were dispensed, they were overlaid with 8 mL of mineral oil. Dishes were made with two different types of oil, heavy and light oil (LifeGuard). The dishes were made one at a time prior to addition of the oil to ensure that there was minimal evaporation. Three independent measurements for each series were conducted. The oil and the media were dispensed at room temperature and were not equilibrated before dish preparation.

### Oxygen measurements

**Figure 1:**
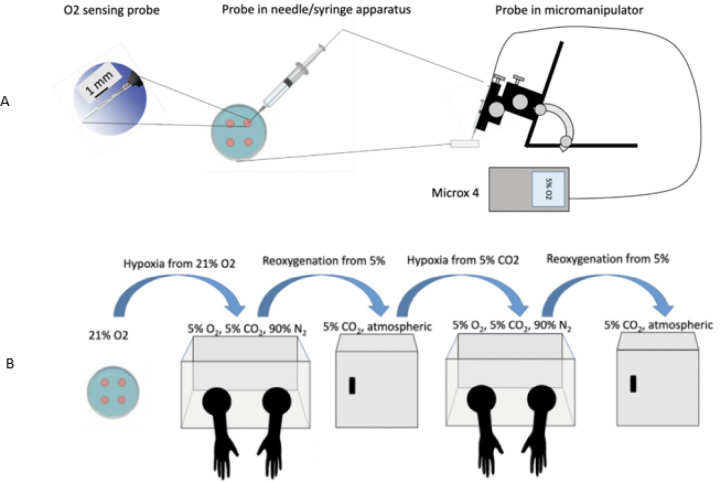
**A:** Diagram of Oxygen Sensing Apparatus. Oxygen concentration monitored using Microx 4 Oxygen Sensor equipped with needle-type probe (Pst-7-flat broken tip, PreSens GmbH, Germany). Probe consists of fiber-optic-line capped with oxygen-sensitive sensor foil that changes its reflective angle with O2 concentration. Fiberoptic line is threaded through a 18 gauge needle and 1 mL tuberculin syringe. A PreSens manual micromanipulator holds the probe in position with sensor foil immersed in media droplet. Sensor was calibrated to 15% w/v sodium sulfite (0% O2) and air saturated water (21% O2). **B**: Diagram of Experimental Timelines. Plates containing oil-embedded media droplets were transferred to hypoxia glovebox incubators (5% O2, 5% CO2, ~90% N2, 37°C, CoyLabs) for 12–18 hours and measured using the Microx 4 as in Figure 1. After transfer and equilibration of the O2 sensor, dish was transferred to a conventional 5% CO2, 37°C incubator and allowed to reoxygenate overnight (12–18 hours). Dish was returned to the Hypoxia Chamber for 16–24 hours and then reoxygenated. O2 measurements were collected every 30 seconds throughout the duration of the experiments.

Changes in oxygen tension were measured every 30 seconds with a Microx4 oxygen meter equipped with a needle-type fiber optic oxygen sensing microprobe (PreSens, GmbH). Each probe was carefully inserted into the center of a culture droplet under mineral oil, avoiding bubble formation or contact with dish plastic. The sensing probes were mounted in a manual micromanipulator to ensure consistent placement of sensor in media droplet (Fig. 1A). Prior to each measurement series the sensor was allowed to equilibrate in each condition for at least five minutes, before transferring dishes to the respective O_2_ conditions.

### Experiment Workflow and Incubation Conditions

Dishes were first created at room temperature in atmospheric oxygen conditions on the bench top. Dishes were then transferred to a hypoxic glove box incubator (CoyLabs, Inc.) set to 5% O_2_, 5% CO_2_, and 37°C. The hypoxic glove box was equipped with a continuous oxygen monitoring system to ensure stable gas composition. The chamber’s oxygen sensor was calibrated to pure nitrogen and atmospheric air prior to each measurement series. The droplet oxygen concentration was measured until O_2_ equilibrated after approximately 12 hours. Following this, plate and probe were then removed to a standard cell culture incubator (NuAire TS Auto Flow CO_2_ water jacketed incubator) equilibrated with atmospheric O_2_ with an actual reading of 18–19% O_2_ and 5% CO_2_ incubator. The single droplets were monitored until saturation was reached (approximately 12 hours). Plates and probes were then transferred back to the hypoxia glove box incubator set to 5% O_2_ and 5% CO_2_. The plates were reoxygenated for a final time for approximately 12 hours until equilibration. (Fig. 1B).

### Replication

The complete experiment was performed three independent times for each oil type, with new dishes, media, and oil preparations. This yielded six hypoxic equilibration curves (three from transitions 21% to 5% O_2_, 5% CO_2_; three from 5% CO_2_ in humidified air) and six reoxygenation curves (5% O_2_, 5% CO_2_ to 21% O_2_, 5% CO_2_ in humidified air) (Fig. 1B).

### Data Analysis

Oxygen concentration (% O_2_) over time was exported from PreSens software. Curves were fitted to a single-phase exponential decay or single-phase association model in GraphPad Prism Software. Data was reported as mean with standard deviation and 95% confidence intervals. Reoxygenation and hypoxic equilibration half times were calculated using least squares fit algorithm in GraphPad Prism. Equilibration curves were compared using extra sum of squares F test with a p-value of < 0.05 considered significant. Reoxygenation dynamics changed quickly for light and heavy oil within the first hour, therefore further discrete points were analyzed at 5-, 10- and 15-minutes intervals using one way ANOVA with corrections for repeated measurements with Dunnett’s multiple comparisons test in GraphPad Prism.

## Results

### Hypoxic equilibration of media

**Figure 2:**
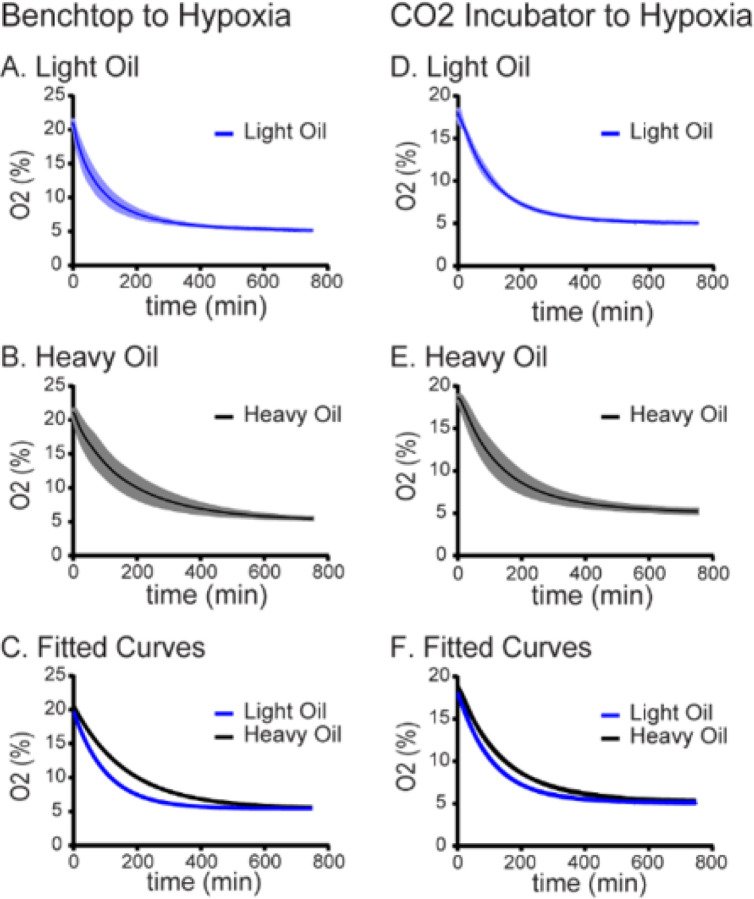
Influence of Atmospheric O2 and Culture Oil Density on the Dynamics of Hypoxic Equilibrium. **A. Hypoxic Equilibration in Light Oil from Atmospheric Conditions.** Oxygen concentration was measured in embryo culture media overlayed with light oil as described in Materials and Methods. The blue line represents the mean oxygen concentration over time after the media was transferred from the benchtop (~20% O_2_) into the hypoxia chamber (~ 5% O_2_, N=3 independent replicates, SE shown in light blue). **B. Hypoxic Equilibration in Heavy Oil from Atmospheric Conditions.** Oxygen concentration was measured in embryo culture media overlayed with heavy oil as described in Panel A. The black line represents the mean oxygen concentration over time following transfer to the hypoxia chamber. (N=3, independent replicates, SE shown in gray). **C. Comparison of Atmospheric-to-Hypoxia Equilibration Curves.** Data from Panels A (blue) and B (black) were fit to single-phase decay curves and compared using extra sum of squares F-test as described in Materials and Methods (p<0.001). **D. Hypoxic Equilibration in Light Oil from Standard Incubator Conditions.** Oxygen concentration was measured in embryo culture media overlayed with light oil. The blue line represents the mean oxygen concentration over time after the media was transferred from a standard incubator (~18–19% O_2_) to a hypoxia chamber (N=3 independent replicates, SE shown in light blue). **E. Hypoxic Equilibration in Heavy Oil from Standard Incubator.** The black line represents the mean oxygen concentration over time after media immersed in heavy oil was transferred from a standard incubator to a hypoxia chamber (N=3 independent replicates, SE shown in gray). **F. Comparison of Incubator-to-Hypoxia Equilibration Curves.** Data from Panels D (blue) and E (black) were fit to single-phase decay curves and compared using extra sum of squares F-test as described in Materials and Methods (p<0.001).

Media droplets immersed in culture oil were equilibrated to hypoxic conditions as described in Material and Methods and illustrated in Fig. 1. When dishes prepared on the benchtop (atmospheric, ~ 21% O2, n = 3) were transferred to the hypoxia chamber, complete equilibration to 5% O_2_ took approximately 12 hours with light oil overlay (Fig. 2A). Equilibration followed a single-phase decay curve, with a plateau of 5.434% O2 and a half-life of 71.03 minutes (CI 95%; 70.23 to 71.84 mins; Table 1). Decay curves for independent replicate experiments using heavy oil immersion (n = 3) demonstrated slower trajectory when transferred from atmospheric oxygen to a hypoxia chamber, with a plateau of 5.440% O2 and a half-life of 116.3 minutes (CI 95%; 114.4 to 118.2, Fig. 2B and Table 1). Comparison of the decay curves using the extra sum of squares F-test demonstrated a statistically significant difference (P < 0.0001) between the light oil (K = 0.009759; CI 95% 0.009648 to 0.00987) and heavy oil preparations (K = 0.005961; 95% CI 0.005865 to 0.006058; Table 1 and Fig. 2C).

Similarly, when equilibrated dishes overlain with light oil were transferred to the hypoxia chamber from a standard humidified 5% CO2 incubator set to atmospheric conditions (n = 3, ~ 18–19% O2), equilibration was also complete in approximately 12 hours, and followed a single-phase decay curve with a plateau of 5.141% O2 and a half-life of 76.76 minutes (CI 95%; 76.49 to 77.03 mins; Fig. 2D and Table 1). When droplets equilibrated in heavy oil were moved from the incubator to the hypoxia chamber (n = 3), oxygen measurements also followed single-phase decay curve with a plateau of 5.311% O2 and a half-life of 98.33 minutes (CI 95%; 97.09 to 99.59, Fig. 2E and Table 1). The equilibration curves for these two series were significantly different (P < 0.0001, Fig. 2F and Table 1). The light oil curve had a K-value of 0.009030 (CI 95%; 0.0089 to 0.0091) while heavy oil had a K-value of 0.007049 (CI 95%; 0.006960 to 0.007139).

### Reoxygenation Dynamics

**Figure 3:**
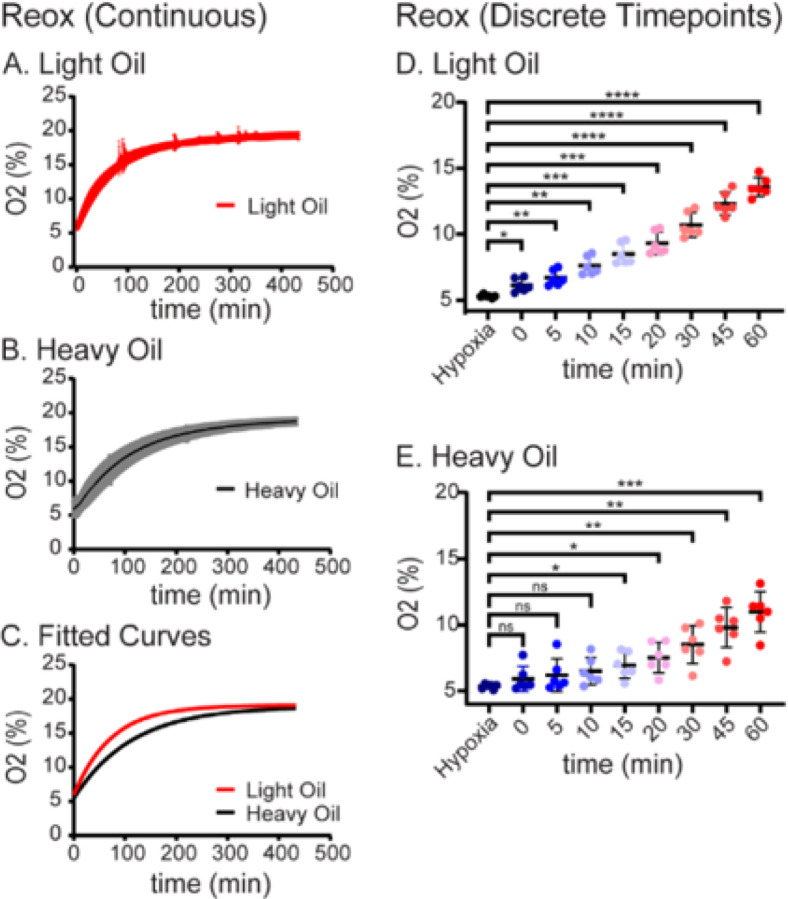
Influence of Oil Density on Dynamics of Reoxygenation. **A. Reoxygenation Dynamics in Light Oil.** Oxygen concentration was measured in embryo culture media overlayed with light oil as described in Materials and Methods. The red line represents the mean oxygen concentration over time after hypoxic media (~5% O_2_) transferred into an atmospheric incubator (~ 18–19% O_2_; N=6 independent replicates, SE shown in light red). **B. Reoxygenation Dynamics in Heavy Oil.** Oxygen concentration was measured in embryo culture media overlayed with heavy oil. The black line represents the mean oxygen concentration over time after the media was transferred into an atmospheric incubator (~ 18–19% O_2_; N=6 independent replicates, SE shown in gray). **C. Comparison of Reoxygenation Curves.** Data from Panel A (light oil, blue) and Panel B (heavy oil, black) was fit to single-phase decay curves as described in Materials and Methods and compared using extra sum of squares F-test as described in Materials and Methods (p<0.001). **D. Reoxygenation at Discrete Time Points in Light Oil.** Average O_2_% measurements were plotted at discrete timepoints for up to 60 minutes following reoxygenation of embryo culture overlain in light oil. Data represents the mean concentration ± 95% confidence intervals compared to Hypoxia using One-Way ANOVA of repeated measurements with Dunnet’s multiple comparison test (* = P < 0.05, ** = P < 0.01, *** = P < 0.001, **** = P < 0.0001). **E. Reoxygenation at Discrete Points in Heavy Oil.** Data represents the mean concentration ± 95% confidence intervals compared to Hypoxia using One-Way ANOVA of repeated measurements with Dunnet’s multiple comparison test (* = P < 0.05, ** = P < 0.01, *** = P < 0.001).

In both light oil and heavy oil media preparations, media was allowed to reach complete reoxygenation for approximately 12 hours. The doubling time for reoxygenation in the light oil was 50.22 minutes (CI 95%; 49.63 to 50.82 minutes) (Table 2). Reoxygenation dynamics of light oil (n = 6) demonstrated a single-phase association curve with K-value of 0.01380 (CI 95%; 0.01364 to 0.01296; Fig. 3A and Table 2). Comparatively, the reoxygenation doubling time for heavy oil was 78.49 minutes (CI 95%; 77.07 to 79.95, Table 2). The reoxygenation of the droplets immersed in heavy oil also demonstrated a single-phase association curve with a K value of 0.008831 (CI 95%; 0.008670 to 0.008993; Table 2 and Fig. 3B). Comparison of reoxygenation curves with the extra sum of squares F test demonstrated significantly slower reoxygenation dynamics for the heavy oil dishes (P < 0.0001; Fig. 3C and Table 2).

Reoxygenation of media overlain by light oil in the humified incubator (~ 18–19% O2) was statistically significant immediately following transfer (t = 0 minutes), with a mean difference of − 0.8330 compared to hypoxic equilibration (CI 95%; −1.487 to − 0.1794) and a p-value of 0.0191 (Fig. 3D and Table 3). The reoxygenation of media under heavy oil was at a slower rate than the light oil with statistically significant reoxygenation occurring 15 minutes after transfer from hypoxia, with a mean difference of − 1.590 (CI 95%; − 2.944 to − 0.2362) and a p-value 0.026 (Fig. 3D and Table 4).

## Discussion

To the best of our knowledge, our study provides the first known, direct quantitation of oxygen equilibration and reoxygenation dynamics in embryo culture media using conditions designed to mimic routine ART laboratory practices. We demonstrated that oxygen exchange is gradual but predictable with equilibration under hypoxia. Our results also demonstrate that reoxygenation can occur rapidly with significant rises in O_2_ within minutes of exposure to ambient air. Additionally, the choice of oil overlay significantly alters these dynamics, with light oil allowing faster equilibration and reoxygenation compared to heavy oil. The influence of oil type impacts buffering capacity and reoxygenation rates, creating a dilemma in oil choice for embryo culture.

The intent of our experimental design was to closely reflect practices in embryology laboratories. During routine *in vitro* fertilization (IVF) procedures, media preparation is frequently performed on the benchtop in atmospheric oxygen, after which plates are transferred into low oxygen incubators to more closely mimic the physiologic environment *in utero*. The first portion of our experiment closely emulated this, as the dishes were prepared on the bench top then moved to a hypoxic incubator (Fig. 1B). Similarly, oocytes and embryos are often removed from the hypoxic incubator for micromanipulation steps such as intracytoplasmic sperm injection (ICSI) or embryo biopsy, briefly exposing them to atmospheric oxygen before being returned to reduced oxygen [21]. Since embryo manipulation often takes place in atmospheric oxygen, to recreate the laboratory environment, the O2 sensor was equilibrated in the destination incubator, dishes were then transferred to a conventional 5% CO2, 37°C incubator and allowed to equilibrate overnight to atmospheric conditions to measure reoxygenation dynamics of embryo culture media (Fig. 1B).

Some ART labs perform their dish preparation in a preequilibrated incubator (18–19% O_2_) then moved into a hypoxic incubator. Therefore, we assessed hypoxia equilibrium when embryo culture media was moved from an atmospheric incubator to a hypoxia chamber (5% O_2_). In our experiment the dishes were then returned to the hypoxia chamber for at least 12-hours and then reoxygenated to mimic the routine handling steps, allowing us to quantify the equilibration dynamics of culture media under realistic laboratory conditions.

In our study we used a hypoxic glove box incubator (hypoxia chamber) maintained at 5% O_2_ which prevented reoxygenation artifacts during the process of manipulating the oxygen probe. This chamber also maintained constant temperature, which can also influence oxygen sensor readings [4]. A primary goal of our study was to mimic culture conditions without inserting the additional influence of embryo metabolism. Our work highlights how fluctuations in environmental oxygen saturation could influence the stability of the microenvironment surrounding embryos. By characterizing the kinetics of oxygen equilibration across different manipulations, our study underscores the potential for transient reoxygenation events to affect embryo culture outcomes. This provides valuable insight into best practices for minimizing oxygen fluctuations, reinforcing the importance of consistent hypoxic culture conditions in optimizing IVF success rates.

Both the light and heavy oil equilibrations followed single-phase decay curves, but the half-life to equilibration in light oil was significantly lower than the half-life for the heavy oil. These results indicate that longer equilibration times should be considered in standard laboratory workflows that use heavy oil. This highlights the impact of oil viscosity and density on oxygen diffusion, which had been suggested in theory but here is directly measured [22].

The result of our study has relevant implications for ART practice. Notably, oxygen concentrations used for embryo culture vary considerably across clinics and regions. Many laboratories continue to culture embryos at atmospheric oxygen (20%) despite recommendations supporting reduced oxygen conditions (5%) to more closely reflect the physiologic reproductive environment [23, 24]. Culturing embryos at elevated oxygen has been shown to cause massive gene deregulation in mice, including genes required for cell growth and gastrulation [25]. Similarly, bovine embryos cultured under elevated oxygen exhibit increased DNA methylation and reduced blastocyst formation rates [25, 26]. Exposure to atmospheric oxygen further enhances the activity of oxygen-dependent enzymes, leading to accelerated ROS generation, heightened oxidative stress, and prolonged embryo development [27, 28]. This dynamic is particularly concerning given the known sensitivity of preimplantation embryos to oxygen fluctuations and the potential for reactive oxygen species (ROS) mediated damage [29]. ROS are often generated *in vitro* and rise if oxygen tensions of the media increases, impairing embryo competence and viability [30] [31]. This can impair embryo competence and viability [30].

Our study has some limitations. First, our kinetic data was derived from a media only system without the influence of embryo metabolism. Embryos may alter oxygen dynamics due to metabolic waste production and cellular oxygen consumption which may accelerate or delay equilibration rates [29]. Investigating oxygen kinetics in the presence of embryos, as well as directly measuring ROS levels, could provide more biologically relevant insights. Furthermore, only one formulation of culture media was tested therefore this data may not be generalizable for all other media types. Further studies would be recommended to compare multiple media types. Additionally, media droplets were pipetted into the dish to mimic standard embryo culture conditions, but only one of the droplets could be measured at one time due the oxygen monitor only having one sensor input. Attempts to measure changes in all four droplets in sequence resulted in equilibration artifacts due to the transition of the sensor between droplets. More consistent readings were achieved by fixing the probe in one droplet with a micromanipulator (Fig. 1A). Incubator space also did not allow for more than one micromanipulator per experiment. Finally, our study did not evaluate the impact of the shift in oxygenation on the embryo itself. It is not yet known if short term shifts in oxygen have an impact on embryo quality.

Taken together, these results argue strongly for minimizing atmospheric exposure during all phases of embryo culture and for implementing hypoxic workstations or isolettes wherever feasible. Moreover, our direct measurements of equilibration kinetics offer embryologists practical guidance on how quickly reoxygenation occurs and how oil overlay choices modulate this process. By defining these parameters, our study provides practical and quantitative benchmarks for reoxygenation kinetics, that contribute to the development of evidence-based best practices aimed at reducing oxygen-related artifacts, safeguarding embryo development, and ultimately improving IVF outcomes.

## Figures and Tables

**Table 1:** Hypoxia Trends

	Light Oil	Heavy Oil
**Atmosphere > 5% O**_**2**_		
Half Life [mins]; (95% CI)	71.03; (70.23 to 71.84)	116.3; (114.4 to 118.2)
K-value; (95% CI)	0.0098; (0.0096 to 0.0098)	0.0060; (0.0059 to 0.0061)
**Atmospheric Incubator > 5% O**_**2**_		
Half Life; (95% CI)	76.76; (76.49 to 77.03)	98.3; (97.09 to 99.59)
K-value; (95% CI)	0.0090; (0.0089 to 0.0091)	0.0071 (0.0070 to 0.0071)

**Table 2:** Reoxygenation Trends

	Light Oil	Heavy Oil
**5% O**_**2**_ **> Atmospheric Incubator**		
Doubling time [mins]; (95% CI)	50.22; (49.63 to 50.82)	78.49; (77.07 to 79.95)
K-value; (95% CI)	0.0138; (0.0136 to 0.0129)	0.0088; (0.0087 to 0.0090)

**Table 3:** Light Oil Reoxygenation Discrete points

Dunnett’s multiple comparisons test	Mean diff.	95.00% CI of diff.	Below threshold	Adjusted P - Value
Hypoxia vs. 0	−0.833	−1.487 to −0.179	Yes	0.0191
Hypoxia vs. 5	−1.385	−2.169 to −0.600	Yes	0.0046
Hypoxia vs. 10	−2.301	−3.279 to −1.324	Yes	0.0012
Hypoxia vs. 15	−3.197	−4.335 to −2.059	Yes	0.0005
Hypoxia vs. 20	−4.009	−5.258 to −2.761	Yes	0.0003
Hypoxia vs. 30	−5.392	−6.751 to −4.033	Yes	<0.0001
Hypoxia vs. 45	−7.004	−8.245 to −5.764	Yes	<0.0001
Hypoxia vs. 60	−8.263	−9.296 to −7.229	Yes	<0.0001

**Table 4:** Heavy Oil Reoxygenation Discrete points

Dunnett’s multiple comparisons test	Mean diff.	95.00% CI of diff.	Below threshold	Adjusted P-Value
Hypoxia vs. 0	−0.563	−1.973 to 0.846	No	0.5646
Hypoxia vs. 5	−0.843	−2.678 to 0.994	No	0.4514
Hypoxia vs. 10	−1.149	−2.604 to 0.306	No	0.1140
Hypoxia vs. 15	−1.590	−2.944 to −0.236	Yes	0.0268
Hypoxia vs. 20	−2.163	−3.770 to −0.555	Yes	0.0153
Hypoxia vs. 30	−3.157	−5.215 to −1.099	Yes	0.0087
Hypoxia vs. 45	−4.459	−6.662 to −2.256	Yes	0.0025
Hypoxia vs. 60	−5.638	−7.860 to −3.417	Yes	0.0009

## Data Availability

All data supporting the findings of this study are available within the paper.
